# Supplementary material for: Reducing tuberculosis transmission by genotype-based contact tracing coupled with public health containment measures: a case study during the COVID-19 pandemic in Taiwan
Source: Microbiol Spectr. 2025 Mar 26;13(5):e02125-24. doi: 10.1128/spectrum.02125-24 (PMC12053905; doi:10.1128/spectrum.02125-24)

**Supplementary Table 1** Distribution of notified confirmed domestic COVID-19 cases in each administrative region and city or county in Taiwan, 2020–2021.

| Regions | No. of cases (%) | | Population | Incidence^*^ | No. of deaths | Mortality^*^ |
| --- | --- | --- | --- | --- | --- | --- |
| **Taipei region** | **12,240** | **(83.7%)** | **7,607,912** | **160.9** | **773** | **10.2** |
| Taipei City | 4,880 | (33.4%) | 2,602,418 | 187.5 | 326 | 12.5 |
| New Taipei City | 6,938 | (47.5%) | 4,030,954 | 172.1 | 416 | 10.3 |
| Keelung City | 319 | (2.2%) | 367,577 | 86.8 | 29 | 7.9 |
| Yilan County | 99 | (0.7%) | 453,087 | 21.9 | 2 | 0.4 |
| Kinmen County | 0 | (0.0%) | 140,597 | 0 | 0 | 0.0 |
| Lienchiang County | 4 | (0.0%) | 13,279 | 30.1 | 0 | 0.0 |
| **Northern region** | **1,510** | **(10.3%)** | **3,833,584** | **39.4** | **46** | **1.2** |
| Taoyuan City | 807 | (5.5%) | 2,268,807 | 35.6 | 27 | 1.2 |
| Hsinchu City | 43 | (0.3%) | 451,412 | 9.5 | 2 | 0.4 |
| Hsinchu County | 111 | (0.8%) | 570,775 | 19.4 | 14 | 2.5 |
| Miaoli County | 549 | (3.8%) | 542,590 | 101.2 | 3 | 0.6 |
| **Central region** | **519** | **(3.5%)** | **4,578,289** | **11.3** | **24** | **0.5** |
| Taichung City | 207 | (1.4%) | 2,820,787 | 7.3 | 8 | 0.3 |
| Changhua County | 274 | (1.9%) | 1,266,670 | 21.6 | 15 | 1.2 |
| Nantou County | 38 | (0.3%) | 490,832 | 7.7 | 1 | 0.2 |
| **Southern region** | **114** | **(0.8%)** | **3,317,276** | **3.4** | **2** | **0.1** |
| Yunlin County | 22 | (0.2%) | 676,873 | 3.3 | 1 | 0.1 |
| Chiayi City | 10 | (0.1%) | 266,005 | 3.8 | 0 | 0.0 |
| Chiayi County | 32 | (0.2%) | 499,481 | 6.4 | 0 | 0.0 |
| Tainan City | 50 | (0.3%) | 1,874,917 | 2.7 | 1 | 0.1 |
| **Kaoping region** | **147** | **(1.0%)** | **3,684,542** | **4.0** | **3** | **0.1** |
| Kaohsiung City | 94 | (0.6%) | 2,765,932 | 3.4 | 1 | 0.0 |
| Pingtung County | 48 | (0.3%) | 812,658 | 5.9 | 0 | 0.0 |
| Penghu County | 5 | (0.0%) | 105,952 | 4.7 | 2 | 1.9 |
| **Eastern region** | **90** | **(0.6%)** | **539,633** | **16.7** | **3** | **0.6** |
| Hualien County | 68 | (0.5%) | 324,372 | 21.0 | 2 | 0.6 |
| Taitung County | 22 | (0.2%) | 215,261 | 10.2 | 1 | 0.5 |
| **Total** | **14,620** | **(100.0)** | **23,561,236** | **62.1** | **851** | **3.6** |

^*^Per 100,000.

**Supplementary Table 2** Changes in mortality rates of TB before and during the COVID-19 pandemic in Taiwan, 2017–2021.

|  | Before the pandemic | | | | During the pandemic | | | | Change^*^  % | Decrease^**^  % |
| --- | --- | --- | --- | --- | --- | --- | --- | --- | --- | --- |
|  | 2017 | 2018 | 2019 | AV | | 2020 | 2021 | AV |  |  |
| Total | 2.2 | 2.1 | 2.3 | 2.2 | | 2.0 | 1.9 | 2.0 | 88.6% | 11.4% |
| Age group (years) |  |  |  |  | |  |  |  |  |  |
| 0-14 | 0 | 0 | 0 | 0.0 | | 0 | 0 | 0.0 |  |  |
| 15-24 | 0 | 0 | 0 | 0.0 | | 0 | 0 | 0.0 |  |  |
| 25-34 | 0.2 | 0 | 0.1 | 0.1 | | 0.1 | 0 | 0.1 | 50.0% | 50.0% |
| 35-44 | 0.1 | 0.2 | 0.1 | 0.1 | | 0.1 | 0.1 | 0.1 | 75.0% | 25.0% |
| 45-54 | 0.6 | 0.6 | 0.4 | 0.5 | | 0.4 | 0.7 | 0.6 | 103.1% | -3.1% |
| 55-64 | 1.6 | 1.2 | 1.6 | 1.5 | | 1 | 0.8 | 0.9 | 61.4% | 38.6% |
| 65+ | 13.3 | 12.8 | 13.3 | 13.1 | | 10.9 | 10 | 10.5 | 79.6% | 20.4% |

Abbreviation: AV, average.

^*^Change = [(Average during the pandemic) / (Average before the pandemic)].

^**^Decrease = (1-Change)

**Supplementary Figure 1a** Epidemic curve of weekly newly notified confirmed COVID-19 cases in Taiwan, 2020-2021.


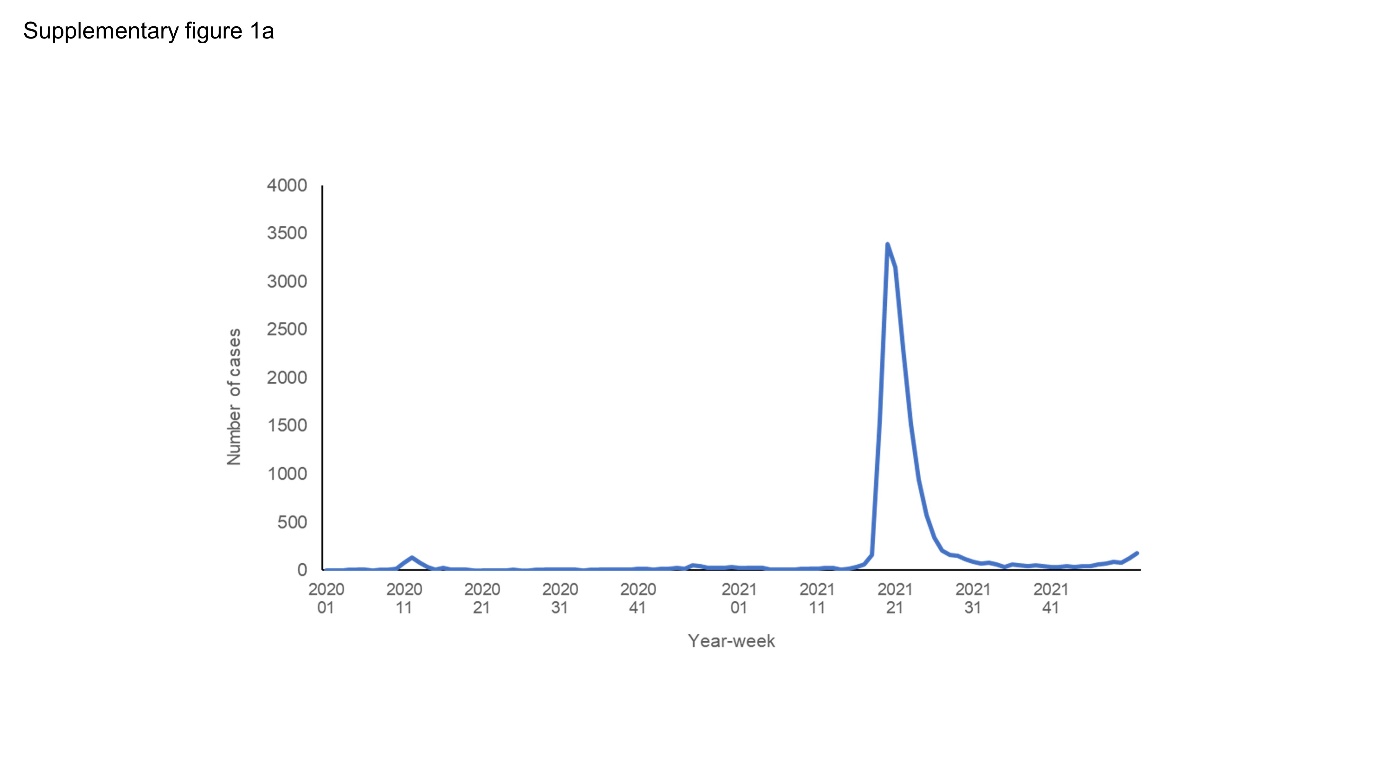


**Supplementary Figure 1b** Geographic distribution of notified confirmed COVID-19 cases in Taiwan, 2020–2021. The six administrative regions were demarcated by a bright yellow line.


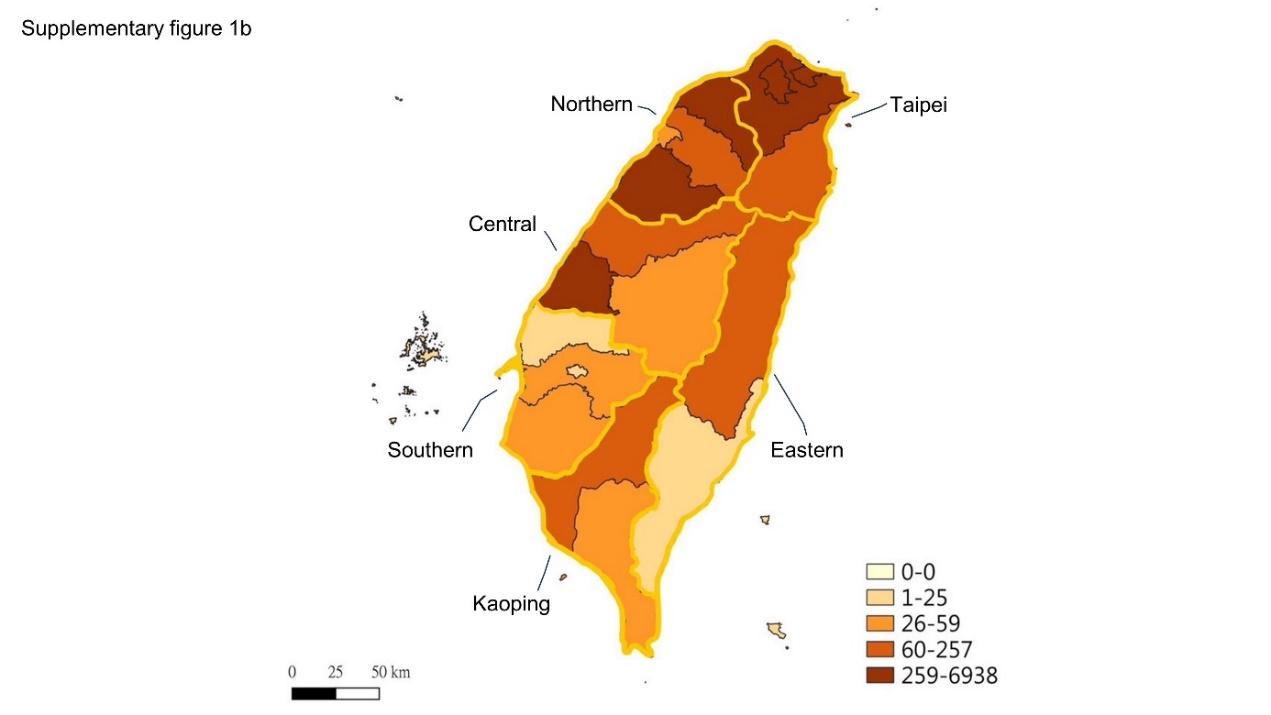

Supplement: Supplemental material — Tables S1 and S2; Fig. S1. [file spectrum.02125-24-s0001.docx]
